# Draft Genome Sequence of Colistin-Only-Susceptible *Pseudomonas aeruginosa* Strain ST235, a Hypervirulent High-Risk Clone in Spain

**DOI:** 10.1128/genomeA.01097-14

**Published:** 2014-10-30

**Authors:** Esther Viedma, Jenifer Villa, Carlos Juan, Antonio Oliver, Fernando Chaves

**Affiliations:** a Servicio de Microbiología Clínica, Hospital Universitario 12 de Octubre, Madrid, Spain; b Spanish Network for Research in Infectious Diseases (REIPI RD12/0015), Instituto de Salud Carlos III, Madrid, Spain; c Servicio de Microbiología and Unidad de Investigación, Hospital Universitario Son Espases, Instituto de Investigación Sanitaria de Palma (IdISPa), Palma de Mallorca, Spain

## Abstract

We report the genome of colistin-only-susceptible *Pseudomonas aeruginosa* strain ST235 (PA_ST235). This isolate was obtained in the setting of an outbreak in a tertiary hospital in Spain. This clone was apparently associated with a significantly higher mortality rate. The ST235 clone also appears to be associated with greater virulence.

## GENOME ANNOUNCEMENT

*Pseudomonas aeruginosa* is an opportunistic pathogen in humans, frequently causing serious infections in immunocompromised and critically ill patients, due to its remarkable ability to combine mutation-driven and horizontally acquired resistance mechanisms (1, 2).

Molecular epidemiology studies, along with a deep genetic investigation of chromosomal and transferable resistance mechanisms, allowed us to describe a highly spread clone ST235 of colistin-only-susceptible (COS) *P. aeruginosa*, simultaneously producing the extended-spectrum β-lactamases (ESBLs) GES-1 and GES-5 in a class 1 integron in a Spanish hospital (3). The ST235 clone is considered a high-risk clone that has been reported in hospital outbreaks worldwide and associated with multidrug resistance patterns by acquisition of different ESBLs and carbapenemases (4–6). However, this clone totally disappeared in our institution and was replaced by another high-risk clone (ST175), which resulted in an endemic situation of multiresistant VIM-2-producing *P. aeruginosa* (7).

The *P. aeruginosa* ST235 genome (PA_ST235) was sequenced using a Roche 454 Junior sequencer. A total of 227,356,902 bp were obtained, providing approximately 26-fold coverage and 476,129 reads. Sequences obtained were used for *de novo* assembly using Newbler Assembler version 3.0 (Roche). The draft genome sequence consists of 130 contigs with an *N*_50_ contig size of 191,815 nucleotides and a total length of 6,930,611 bp. Sequences were annotated using the Rapid Annotation Using Subsystem Technology (RAST) server (8). A total of 6,539 coding DNA sequence (CDS) genes and 60 tRNAs were detected. This approach highlighted the presence of up to 136 genes related to antibiotic and antiseptic resistance. The verification of the class 1 integron harboring tandem duplication of GES1/GES5, as previously reported in this clone (3), was performed with BLAST (http://www.ncbi.nlm.nih.gov/BLAST). Several chromosomal mutations and proteins involved in antibiotic and antiseptic resistance, including many of those previously reported for other lineages, were also detected (9). Furthermore, a total of 62 related phage and prophage elements were detected. The ST235 clone, besides its wide dissemination, appears to be associated with greater virulence (3, 10). PA_ST235 whole genome sequencing revealed the presence of type III secretion system cytotoxins ExoU and ExoY; in particular, ExoU has been associated with increased virulence (10). This strain was not a carrier of any plasmid as occurred with VIM-2-producing the *P. aeruginosa* ST175 clone (11). A genomic comparison of both clones performed with the RAST server revealed that no major differences in resistance mechanisms exist, as previously reported for each of the clones (3, 7). A few genes associated with resistance and virulence were detected in the PA_ST235 clone but not in the ST175 clone: ExoU protein, DedA protein, MacA protein (macrolide-specific efflux) or YdhE/NorM (multidrug and toxin extrusion [MATE] family efflux pump). A comprehensive genomic comparative analysis of the two clones, along with other sporadic and successful clones, might shed some hypotheses on the genetic factors determining clonal success and virulence, and thus establish new containment measures against the spread of this type of clone.

### Nucleotide sequence accession number.

This whole-genome shotgun project has been deposited at DDBJ/EMBL/GenBank under the accession number JNHD00000000.
